# Induction of amylase and protease as antibiofilm agents by starch, casein, and yeast extract in *Arthrobacter* sp. CW01

**DOI:** 10.1186/s12866-021-02294-z

**Published:** 2021-08-23

**Authors:** Jeffrine Solihin, Diana Elizabeth Waturangi, Tresnawati Purwadaria

**Affiliations:** 1 Department of Biotechnology, Faculty of Biotechnology, Atma Jaya Catholic University of Indonesia, 12930 Jakarta, Indonesia; 2 Master Biotechnology Program, Faculty of Biotechnology, Atma Jaya Catholic University of Indonesia, 12930 Jakarta, Indonesia

**Keywords:** *Arthrobacter*, amylase, protease, antibiofilm, *P. aeruginosa*, *S. aureus*, starch, casein, yeast extract

## Abstract

**Background:**

In unfavourable environment, such as nutrient limitation, some bacteria encased themselves into a three dimensional polymer matrix called biofilm. The majority of microbial infections in human are biofilm related, including chronic lung, wound, and ear infections. The matrix of biofilm which consists of extracellular polymeric substances (EPS) causes bacterial colonization on medical implanted device in patients, such as catheter and lead to patient’s death. Biofilm infections are harder to treat due to increasing antibiotic resistance compared to planktonic microbial cells and escalating the antibiotic concentration may result into *in vivo* toxicity for the patients. Special compounds which are non-microbicidal that could inhibit or destroy biofilm formation are called antibiofilm compounds, for example enzymes, anti-quorum sensing, and anti-adhesins. *Arthrobacter* sp. CW01 produced antibiofilm compound known as amylase. This time our preliminary study proved that the antibiofilm compound was not only amylase, but also protease. Therefore, this research aimed to optimize the production of antibiofilm agents using amylase and protease inducing media. The five types of production media used in this research were brain heart infusion (BHI) (Oxoid), BHI with starch (BHIS), casein with starch (CS), yeast extract with starch (YS), and casein-yeast extract with starch (CYS). Biofilm eradication and inhibition activities were assayed against *Pseudomonas aeruginosa* (ATCC 27,853) and *Staphylococcus aureus* (ATCC 25,923).

**Results:**

The results showed that different production media influenced the antibiofilm activity. Addition of starch, casein and yeast extract increased the production of amylase and protease significantly. Higher amylase activity would gradually increase the antibiofilm activity until it reached the certain optimum point. It was shown that crude extracts which contained amylase only (BHI, BHIS and YS) had the optimum eradication activity against *P. aeruginosa* and *S. aureus* biofilm around 60–70 %. Meanwhile, CS and CYS crude extracts which contained both amylase and protease increased the biofilm eradication activity against both pathogens, which were around 70–90 %.

**Conclusions:**

It was concluded that the combination of amylase and protease was more effective as antibiofilm agents against *P. aeruginosa* and *S. aureus* rather than amylase only.

## Background

Bacteria do not always live in planktonic form. In some conditions, the bacteria would attach into a surface and encased themselves in a three dimensional polymer matrix called biofilm as a mechanism to survive harsh living environment. They can switch between planktonic form and biofilm form. According to National Institute of Health, more than 80 % microbial infections are biofilm related, including chronic lung, wound and ear infections. Biofilm formation causes bacterial colonization on medical implanted device in patients, such as catheter and lead to the patient’s death. These kinds of infections are hard to diagnose and treat because of increasing antibiotic resistance in biofilm compared to planktonic cells [1–3].

Previous studies had shown that *Arthrobacter* sp. CW01 produced an antibiofilm compound in the form of amylase enzyme. The crude antibiofilm extract of this bacterium has LC_50_ > 1000 ppm based on brine shrimp lethality test, indicating that it was not toxigenic [4]. As enzymes are rapidly biodegradable, they are more eco-friendly. Therefore, the application of enzyme as antibiofilm compound to combat clinical infections would be an attractive strategy [5].

In order to achieve high yield of amylase and protease production, optimization of chemical composition of culture medium is an important factor. The medium must contain carbon, nitrogen and mineral salts. Carbon is needed to synthesize the bacterial cells. Nitrogen source is needed as the building blocks to synthesize amino acids. Since amylase and protease are proteins, the right choice of nitrogen source may stimulate the secretion of the enzymes. Some mineral salts are also known to trigger the production of both enzymes and their cofactors. Amylase is an inducible enzyme. The presence of starch as sole carbon source in the production medium is known to trigger amylase production. However, it is also known that amylase production is affected by catabolite repression by starch hydrolytic products such as glucose and maltose [6, 7].

The aim of this research is to optimize the production of antibiofilm agents from *Arthrobacter* sp. CW01 using amylase and protease inducing media.

## Results

### Screening for antibiofilm agent production media

**Fig. 1:**
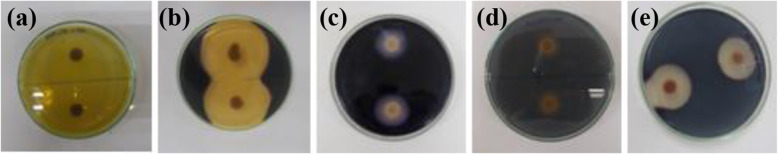
Clearance zone of hydrolytic assay from (**A**) BHI, (**B**) BHIS, (**C**) CS, (**D**) YS, and (**E**) CYS agar production media

After the addition of iodine to the production agar media, BHIS exhibited the largest starch hydrolytic zone (Fig. 1). YS and CS were the bottom two media with the smallest hydrolytic zones. However, the combination of both casein and yeast extract in CYS medium showed an increase in the diameter of the hydrolytic zone, almost 1.72-fold higher than its own constituent components, which were casein and yeast extract (Table 1).

**Table 1:** Hydrolytic zone of the production agar media

Media	Colony diameter (cm)	Clearance zone diameter (cm)	Hydrolytic zone (cm)
BHI	1.35	0.00	0.00
BHIS	1.55	4.30	2.75
CS	1.25	2.55	1.30
YS	1.35	2.55	1.20
CYS	1.05	3.20	2.15

*BHI represented brain heart infusion and used as negative control; BHIS represented brain heart infusion and 1 % soluble starch; CS represented casein, mineral salts and 1 % soluble starch; YS represented yeast extract, mineral salts and 1 % soluble starch; CYS represented casein, yeast extract, mineral salts and 1 % soluble starch

### Determination of amylase activity

The highest amylase enzyme activity of the crude extract was from BHIS at 0.39 IU/mL and the highest specific activity was CYS crude extract at 2.88 IU/mg (Table 2). BHIS crude extract exhibited almost 5-fold higher enzyme activity rather than BHI. Furthermore, all of the crude extracts from simple media (CS, YS and CYS) had higher amylase activities than BHI crude extract. All of the crude extracts from simple media also showed higher specific enzyme activities rather than the complex media (BHI and BHIS).

**Table 2:** Enzyme activity and specific activity for amylase

Media	Enzyme activity (IU/mL)	Specific activity (IU/mg)
BHI	0.08^a^	0.15^a^
BHIS	0.39^d^	0.33^a^
CS	0.18^b^	1.81^c^
YS	0.15^b^	1.11^b^
CYS	0.22^c^	2.88^d^

*Different subscript characters showed that the data were significantly different from other treatments in the same column (*P* < 0.05); BHI represented brain heart infusion; BHIS represented brain heart infusion and 1 % soluble starch; CS represented casein, mineral salts and 1 % soluble starch; YS represented yeast extract, mineral salts and 1 % soluble starch; CYS represented casein, yeast extract, mineral salts and 1 % soluble starch

### Determination of protease activity

Only two of the production media (CS and CYS) showed the presence of protease in their crude extracts (Table 3).

**Table 3:** Enzyme activity and specific activity for protease

Media	Enzyme activity (IU/mL)	Specific activity (IU/mg)
BHI	ND^a^	ND^a^
BHIS	ND^a^	ND^a^
CS	1.86^b^	18.74^b^
YS	ND^a^	ND^a^
CYS	5.01^c^	64.19^c^

*Different subscript characters showed that the data were significantly different from other treatments in the same column (*P* < 0.05); ND represented as not detected; BHI represented brain heart infusion; BHIS represented brain heart infusion and 1 % soluble starch; CS represented casein, mineral salts and 1 % soluble starch; YS represented yeast extract, mineral salts and 1 % soluble starch; CYS represented casein, yeast extract, mineral salts and 1 % soluble starch

### Determination of protein content

**Fig. 2:**
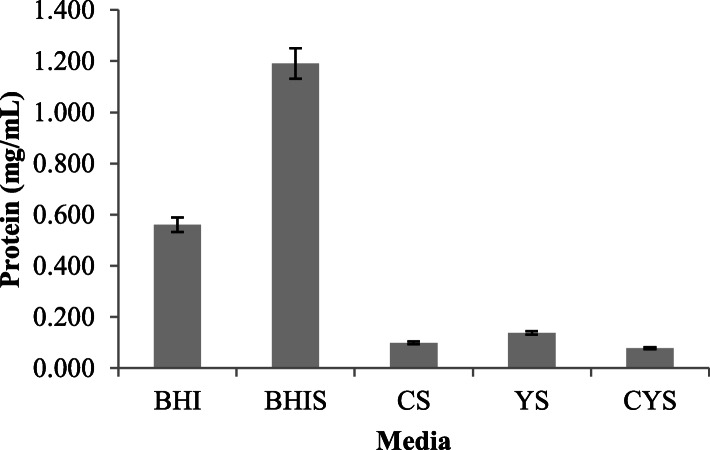
Total protein concentration of the crude extracts

BHIS and CYS crude extracts showed the highest and lowest protein content respectively at 1.19 mg/mL and 0.08 mg/mL (Fig. 2). Based on the data that were already obtained, the crude extracts from the complex media had higher protein content than simple media. Proteins have already consisted in the base formulation of the complex media (BHI and BHIS), with protease peptone as one of the main composition of the original Brain Heart Infusion (Oxoid). Thus, the result also showed that simple media had lower protein content (CS, YS, and CYS).

### Antibacterial activity test

**Fig. 3:**
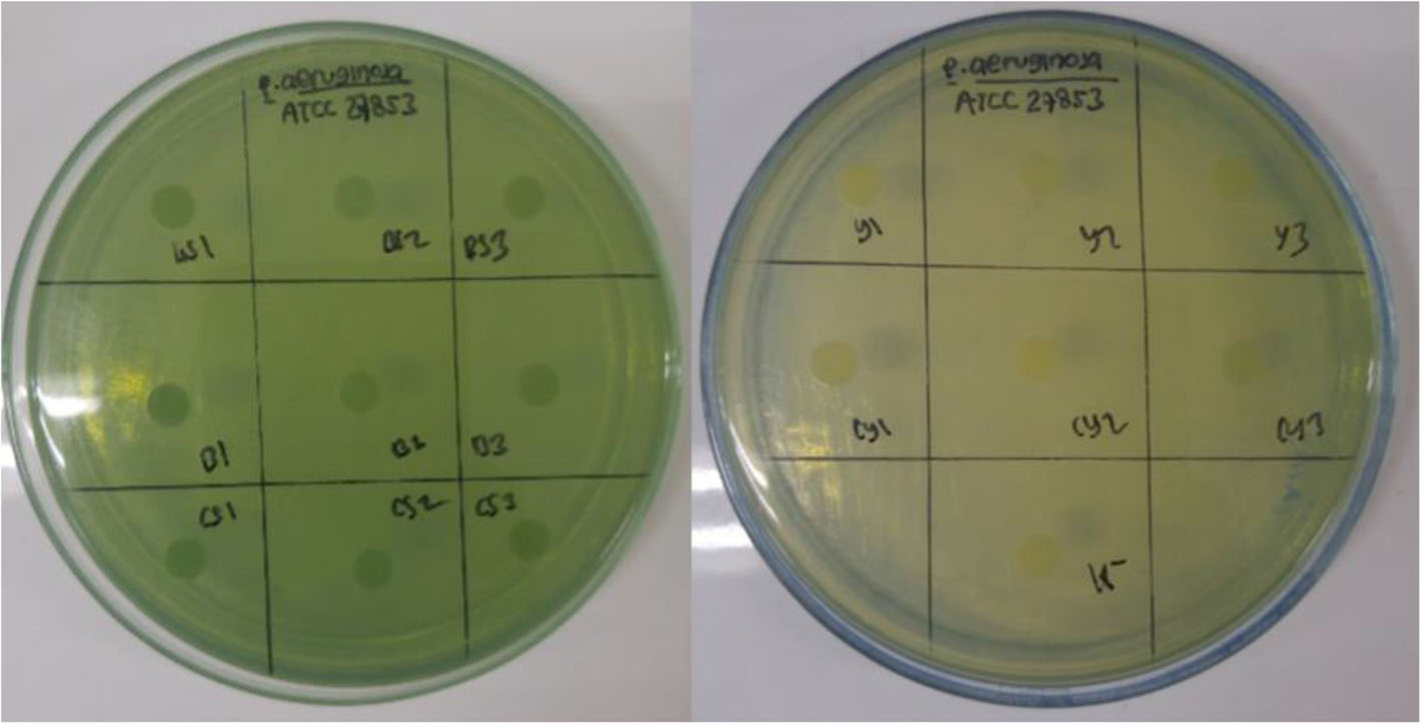
Antibacterial activity against *P. aeruginosa.* BS: Brain Heart Infusion + Starch (BHIS), B: Brain Heart Infusion (BHI); CS: Casein + Starch, Y: Yeast Extract + Starch; CY: Casein, Yeast Extract + Starch; K-: Negative Control. The antibacterial activity from each extract was tested 3 times

**Fig. 4:**
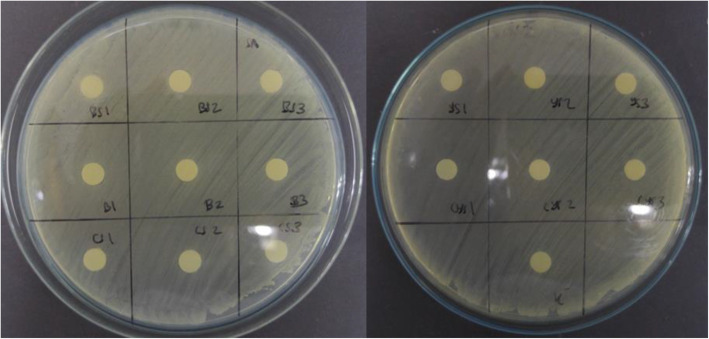
Antibacterial activity against *S. aureus.* BS: Brain Heart Infusion + Starch (BHIS), B: Brain Heart Infusion (BHI); CS: Casein + Starch, Y: Yeast Extract + Starch; CY: Casein, Yeast Extract + Starch; K-: Negative Control. The antibacterial activity from each extract was tested 3 times

Based on the blank disc diffusion test, none of the crude extracts produced clear inhibition zone (Table 4). Those results indicated that none of the enzymes (amylase and protease) in the crude extracts had antibacterial activity against *P. aeruginosa* and *S. aureus* (Figs. 3 and 4).

**Table 4:** Antibacterial activity test

Supernatant	*P. aeruginosa*	*S. aureus*
BHI	-	-
BHIS	-	-
CS	-	-
YS	-	-
CYS	-	-
Ampicillin	-	+
Tetracyclin	+	+
Trimethoprim	-	+
Kanamycin	-	+

*Antibiotics used as control; positive sign (+) represented antibacterial activity; negative sign (-) represented no antibacterial activity

### Biofilm eradication activity assay

**Fig. 5:**
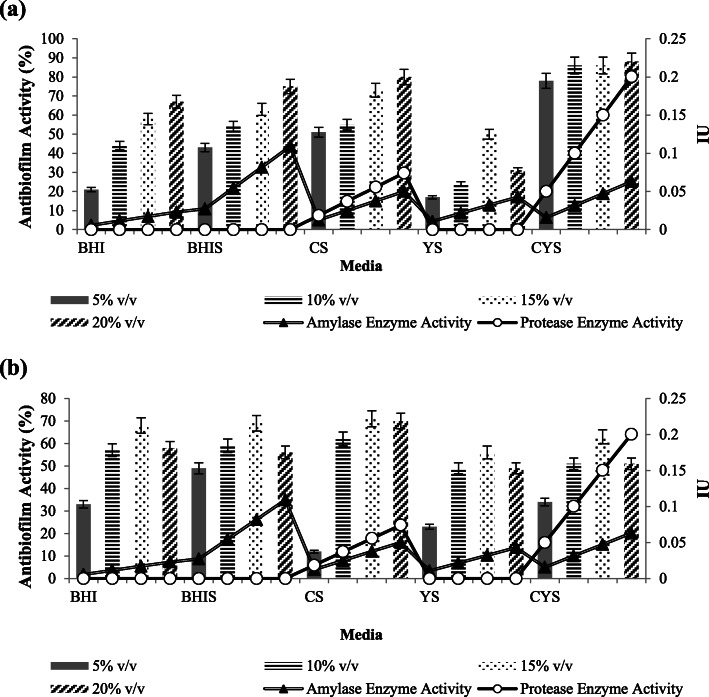
Biofilm eradication activity against (**A**) *P. aeruginosa* and (**B**) *S. aureus.* Amylase enzyme activity was multiplied by 7 times

In general, crude extracts with 20 % v/v concentration from all production media except YS, showed the highest *P. aeruginosa* biofilm eradication activity. The eradication activities of crude extracts from each different production media were significantly different (Tables 5 and 6). The obtained results also revealed that crude extracts with high amylase enzyme activity, such as BHIS, were not always accompanied by the high destruction activity of *P. aeruginosa* biofilm (Fig. 5).

**Table 5:** Homogenous subsets analysis of the crude extracts concentrations in eradicating *P. aeruginosa* biofilm

	Concentration	N	Subset		
			1	2	3
Tukey HSD^a,b^	5 %	45	0.42111		
	10 %	45		0.52776	
	15 %	45			0.65802
	20 %	45			0.68144
	Sig.		1.000	1.000	0.365

Analysis using Tukey’s Honest Significant Difference (HSD) and based on observed means. The error term is Mean Square (Error) = 0.005; (a) Uses Harmonic Mean Sample Size = 45.000; (b) Alpha = 0.05

**Table 6:** Homogenous subsets analysis of the crude extracts media types in eradicating *P. aeruginosa* biofilm

	Media	N	Subset				
			1	2	3	4	5
Tukey HSD^a,b^	YS	36	0.30481				
	BHI	36		0.47425			
	BHIS	36			0.58831		
	CS	36				0.64686	
	CYS	36					0.84619
	Sig.		1.000	1.000	1.000	1.000	1.000

Analysis using Tukey’s Honest Significant Difference (HSD) and based on observed means. The error term is Mean Square (Error) = 0.005; (a) Uses Harmonic Mean Sample Size = 36.000; (b) Alpha = 0.05

Increasing the amylase activity would gradually increase the antibiofilm activity until it reached the maximum point. It was shown that crude extracts which contained amylase only (BHI, BHIS and YS) had maximum eradication activity against *P. aeruginosa* biofilm around 60–70 %. From all of the data obtained, protease enzyme also allegedly contributed to the destruction of *P. aeruginosa* biofilm. Crude extracts which contained both amylase and protease (CS and CYS) increased the destruction activity to 80–90 % (Fig. 5).

Among the four different concentration of crude extracts used for destroying *S. aureus* biofilm, the antibiofilm activity of 10 and 20 % v/v of concentration were not significantly different (Tables 7 and 8). Crude extracts with 15 % v/v of concentration were the optimum concentrations for eradicating *S. aureus* biofilm. Those data proved that increasing amylase or protease activities by adding the crude extract concentrations was not always accompanied by the increase of antibiofilm activity. Overall, the crude extract from CYS with 15 % v/v of concentration had the highest antibiofilm activity at 71.27 % (Fig. 5).

**Table 7:** Homogenous subsets analysis of the crude extracts concentrations in eradicating *S. aureus* biofilm

	Concentration	N	Subset		
			1	2	3
Tukey HSD^a,b^	5 %	45	0.30193		
	10 %	45		0.55293	
	20 %	45		0.56778	
	15 %	45			0.65162
	Sig.		1.000	0.674	1.000

Analysis using Tukey’s Honest Significant Difference (HSD) and based on observed means. The error term is Mean Square (Error) = 0.004; (a) Uses Harmonic Mean Sample Size = 45.000; (b) Alpha = 0.05

**Table 8:** Homogenous subsets analysis of the crude extracts media types in eradicating *S. aureus* biofilm

	Media	N	Subset		
			1	2	3
Tukey HSD^a,b^	YS	36	0.44289		
	CYS	36		0.49656	
	BHI	36		0.53647	
	CS	36		0.53658	
	BHIS	36			0.58033
	Sig.		1.000	0.056	1.000

Analysis using Tukey’s Honest Significant Difference (HSD) and based on observed means. The error term is Mean Square (Error) = 0.004; (a) Uses Harmonic Mean Sample Size = 36.000; (b) Alpha = 0.05

### Biofilm inhibition activity assay

**Fig. 6:**
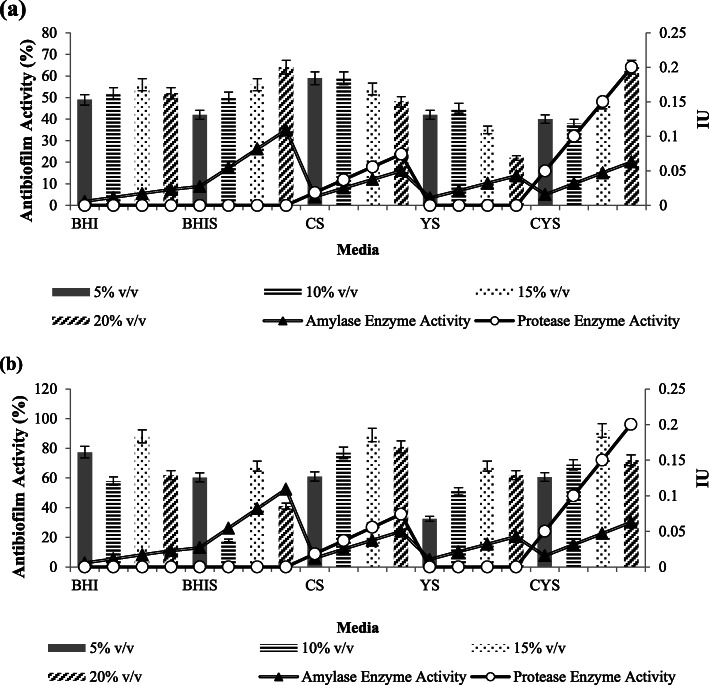
Biofilm inhibition activity against (**A**) *P. aeruginosa* and (**B**) *S. aureus.* Amylase enzyme activity was multiplied by 7 times

Besides showing the best eradication activity against *P. aeruginosa* biofilm, 20 % v/v of crude extract from CYS additionally showed the highest *P. aeruginosa* biofilm inhibition activity at 64.28 % (Fig. 6). Relatively, there was not any significant difference in the antibiofilm activity for the inhibition of *P. aeruginosa* biofilm between the 4 different kinds of crude extract concentrations (Tables 9 and 10). It was also shown that increasing amylase or protease enzyme activities also were not always followed by the increasing biofilm inhibition activities, as shown from CS crude extracts which had declining activities from 10 % v/v to 20 % v/v (Fig. 6).

**Table 9:** Homogenous subsets analysis of the crude extracts concentrations in inhibiting *P. aeruginosa* biofilm

	Concentration	N	Subset
			1
Tukey HSD^a,b^	5 %	45	0.46318
	10 %	45	0.48989
	15 %	45	0.49496
	20 %	45	0.49973
	Sig.		0.159

Analysis using Tukey’s Honest Significant Difference (HSD) and based on observed means. The error term is Mean Square (Error) = 0.007; (a) Uses Harmonic Mean Sample Size = 45.000; (b) Alpha = 0.05

**Table 10:** Homogenous subsets analysis of the crude extracts media types in inhibiting *P. aeruginosa* biofilm

	Media	N	Subset		
			1	2	3
Tukey HSD^a,b^	YS	36	0.35881		
	CYS	36		0.47444	
	BHI	36		0.52153	0.52153
	BHIS	36			0.52981
	CS	36			0.55011
	Sig.		1.000	0.117	0.586

Analysis using Tukey’s Honest Significant Difference (HSD) and based on observed means. The error term is Mean Square (Error) = 0.007; (a) Uses Harmonic Mean Sample Size = 36.000; (b) Alpha = 0.05

Crude extract with the concentration of 15 % v/v was the best concentration to inhibit the formation of *S. aureus* biofilm (Fig. 6). The crude extracts from BHI, CS and CYS with the concentration of 15 % v/v showed high biofilm inhibition activity against *S. aureus*, which were around 88 %. On the other hand, BHIS showed lower inhibition activity rather than BHI. The statistical analysis of BHI and BHIS crude extracts inhibition activity against *S. aureus* also showed that the inhibition activity against the biofilm of *S. aureus* was significantly reduced by the addition of starch in the media (Tables 11 and 12).

**Table 11:** Homogenous subsets analysis of the crude extracts concentrations in inhibiting *S. aureus* biofilm

	Concentration	N	Subset		
			1	2	3
Tukey HSD^a,b^	10 %	45	0.54624		
	5 %	45	0.58427		
	20 %	45		0.64022	
	15 %	45			0.81164
	Sig.		0.149	1.000	1.000

Analysis using Tukey’s Honest Significant Difference (HSD) and based on observed means. The error term is Mean Square (Error) = 0.007; (a) Uses Harmonic Mean Sample Size = 45.000; (b) Alpha = 0.05

**Table 12:** Homogenous subsets analysis of the crude extracts media types in inhibiting *S. aureus* biofilm

	Media	N	Subset			
			1	2	3	4
Tukey HSD^a,b^	BHIS	36	0.47089			
	YS	36		0.53492		
	BHI	36			0.71411	
	CYS	36			0.73594	0.73594
	CS	36				0.77211
	Sig.		1.000	1.000	0.810	0.371

Analysis using Tukey’s Honest Significant Difference (HSD) and based on observed means. The error term is Mean Square (Error) = 0.007; (a) Uses Harmonic Mean Sample Size = 36.000; (b) Alpha = 0.05

## Discussion

Amylase production media screening proved that BHI with the addition of 1 % starch generated the highest amylase production (Table 2). BHI was a complex medium with rich nutrients that can be used for bacterial growth. The availability of ready-to-use nutrients was used for biomass production [8]. Amylase was known as an inducible enzyme. Hence, the addition of starch contributed to the enzyme synthesis [6]. The result also showed that the combination of both casein and yeast extract in the medium (CYS) also generated decent amount of amylase (Table 2). Nitrogen source was an important factor in amylase production. Casein and yeast extract were organic nitrogen sources. Unlike inorganic nitrogen sources, they did not act solely as a source of nitrogen but also supply vitamins, minerals and accessory growth factors in the growth media. Consequently, the combinations of those factors leaded to the increased bacterial growth and at the same time also triggered enzyme production [9].

Crude extracts from BHI had a low amylase enzyme activity, even lower than all of the simple media that were used (Table 2). BHI was the only media without starch. Haseltine et al. 1996 reported that in the archaea *Sulfolobus solfataricus*, α-amylase was produced constitutively in low level in the absence of inducer, such as starch. It is indicated that *Arthrobacter* CW01 also produced amylase in the absence of starch in low level [10]. Most of bacteria preferred to use the simplest available carbohydrate first, such as glucose rather than starch [6]. In CS, YS, and CYS media, starch was the only available carbohydrate. So, in order to sustain cell growth, the bacteria would synthesize amylase to break down starch into glucose that would be ready to use.

Nevertheless, the addition of starch escalated amylase enzyme activity in nutrient-rich media rather than simpler media. This can be seen on the fact that BHIS crude extract had higher amylase activity than CS, YS and CYS (Table 2). This phenomenon may be caused by glucose depletion in BHIS itself. After all of the available glucose in BHIS was used, the number of *Arthrobacter* CW01 cells was high and these bacteria would finally use the starch that was available as the carbon source to continue their cell growth. Higher biomass would generate higher products as amylase was a primary metabolite, thus explaining why BHIS had higher amylase enzyme activity rather than the simpler media [6, 8].

The production media used to produce antibiofilm agents had two major differences, which were rich media and supplemented mineral salt media (Table 13). Li et al. 2014 reported that those distinctive media resulted in the different metabolic potential of *Esherichia coli* strain BL21 [11]. As the mineral salt media were supplemented with casein and yeast extract, it was assumed that they were used and metabolized by *Arthrobacter* CW01. Based on those assumptions, further test was done in the form of proteolytic enzyme test. Two of the crude extracts, CS and CYS were proven to bear proteolytic enzymes (Table 3). Other than aiding amylase production, it was reported that casein and yeast extract also served as nitrogen sources in protease production [9].

**Table 13:** Composition of various antibiofilm agent production media

Medium		Composition (g L^-1^)
BHI	Brain heart infusion (Oxoid) 37.0; Bacteriological agar (Oxoid) 20.0.	
BHIS	Brain heart infusion (Oxoid) 37.0; Soluble starch (Merck) 10.0; Bacteriological agar (Oxoid) 20.0.	
CS	Casein (Sigma-Aldrich) 0.3; Soluble starch 10.0 (Merck); Bacteriological agar (Oxoid) 20.0; KNO_3_ 2.0; NaCl 2.0; KH_2_PO_4_ 2.0; MgSO_4_.7H_2_O 0.05; CaCO_3_ 0.02; FeSO_4_.7H_2_O 0.01 [21].	
YS	Yeast extract 1.0 (Oxoid); Soluble starch (Merck) 10.0; Bacteriological agar (Oxoid) 20.0; NaNO_3_ 1.2; KH_2_PO_4_ 3.0; K_2_HPO_4_ 6.0; MgSO_4_.7H_2_O 0.2; CaCl_2_.2H_2_O 0.05; MnSO_4_.7H_2_O 0.01; ZnSO_4_.7H_2_O 0.001 [22].	
CYS	Casein (Sigma-Aldrich) 0.3; Yeast extract 1.0 (Oxoid); Soluble starch 10.0 (Merck); Bacteriological agar (Oxoid) 20.0; KNO_3_ 2.0; NaCl 2.0; KH_2_PO_4_ 2.0; MgSO_4_.7H_2_O 0.05; CaCO_3_ 0.02; FeSO_4_.7H_2_O 0.01 [21].	

Casein was a better nitrogen source rather than gelatin, yeast extract and beef extract for protease production in *Bacillus pumilus* SG2. This was because casein served as protease-specific inducer, but high concentration of casein could act as substrate repressor. Yeast extract was rich in amino acids mixture, vitamins and growth stimulating compounds which tend to promote cell growth rather than enzyme synthesis and usually used as growth supplement in growth media [8, 9, 12]. This could be seen in *Arthrobacter* CW01 colonies that were grown in YS agar medium (minimum media with yeast extract) had bigger colonies diameter size compared to CS agar medium (minimum media with casein) (Table 1). The difference in colonies diameter size further proved that yeast extracts stimulated cell growth. Those arguments explained why the utilization of casein resulted in better protease activity than yeast extract only and the combination of those two escalated the protease activity.

Another interesting point was that rich media, such as BHI and BHIS, did not induce protease production (Table 3). Michotey and Blanco 1994 reported that there seemed to be an inverse relationship between the ability of *Arthrobacter aureus* to grow and its protease synthesis. Protease production decreased dramatically when *Arthrobacter* was grown on media with high concentrations of casamino acids. Even with low concentrations of casamino acids, the presence of abundant carbon source such as glucose, succinate, citrate, sucrose or glycerol resulted in the reduction of protease production. Protease production in *Arthrobacter* reached the maximum level during late the exponential phase of growth and was not regulated by nutrient starvation [13].

Different production media caused a variance in the total protein content of the crude extracts (Fig. 2). Enzyme activity was associated with protein molecules [14]. Consequently, crude extract with the highest amylase enzyme activity (BHIS) had the highest total protein content. However, this was not supposedly true to other media. Crude extract from BHI had the second highest total protein content even though it had the lowest amylase enzyme activity and did not contain protease. The high protein content in BHI crude extracts may be due to the soluble nutrients in BHI which was derived from organ tissues. Organ tissues contained rich nutrients, especially amino acids [8]. Since the total protein content was measured before protein purification, protein other than the enzymes, such as residual amino acids, may still be present in the supernatant and measured. Michotey and Blanco 1994 reported that during enzyme production, one of *Arthrobacter* species (*A. aureus*), also secreted other extracellular proteins besides the enzyme [13]. On the other hand, low protein content of the crude extracts from simple media (CS, YS and CYS) may be due to the usage of the available protein source in the media to build up the biomass first, consequently reducing the amount of total protein in the supernatant [15]. However, it was noted that the increase of biomass was not always followed by the increase of enzyme production [10].

Based on the antimicrobial assay (Table 4), all of the crude extracts did not show clear zone of inhibition against *P. aeruginosa* and *S. aureus*. These data implied that the enzymes had no bactericidal activity and only posed antibiofilm activity. Thus, the main cause of the lowered biofilm absorbance values after crude extract additions was the disruption of the biofilm EPS components and not by the decreasing number of the viable bacteria that formed the biofilm structure [5]. Even though Actinomycetes was known for antibiotic production, in this case *Arthrobacter* CW01 did not produce any microbicidal compounds with all different kinds of the production media used. This was due to the production of amylase that will hydrolyze the starch into glucose and resulted in high level of glucose in the environment. It was reported that glucose abundance interfered with the formation of antibiotics, which were mainly secondary metabolites. Another distinctive feature of secondary metabolism was its association with low bacterial growth level [16].

From the biofilm eradication activity result (Fig. 5), the antibiofilm compound had higher destruction activity against *P. aeruginosa* rather than *S. aureus* in general. One of the possible explanations was the high degree of heterogeneity of EPS among different bacterial species; therefore the compositions of biofilm structure will be different. Hydrolase-group of antibiofilm compounds such as amylase and protease were allegedly responsible to the dispersal of mature biofilm by degrading the physical integrity of EPS structure and digesting slime layers of the biofilm [5].

The increase of antibiofilm concentration in treatment enhanced the destruction activity for *P. aeruginosa* biofilm (Fig. 5). This may be due to the dose-dependent effect of the antibiofilm compound. Higher crude extracts concentration would result in the higher presence of the bioactive compound [17]. On the contrary, the optimum antibiofilm concentration for destroying *S. aureus* was 15 % (v/v) of concentration and increasing the concentration resulted in a declining in number (Fig. 5). The possibility of this incident may be caused by the fact that each enzyme had its own optimum concentration for each substrate and the presence of inhibitors [14].

Other than disrupting mature biofilm, hydrolases were reported to be responsible for the hydrolysis of the substrate involved in bacterial adhesion. Thus, the enzymes prevented bacterial adhesion that resulted in the inhibition of biofilm formation. Kalpana et al. 2012 reported that the use of amylase alone was reported to inhibit biofilm formation of *S. aureus*, *P. aeruginosa* and *V. cholerae* [5]. However, the biofilm inhibition activity of *S. aureus* showed an interesting result (Fig. 6). Crude extract from BHIS had lower biofilm inhibition activity than BHI even though it had higher amylase activity. One of the possible reasons was that the BHIS crude extract still contained glucose as the hydrolysis product from starch. Lim et al. 2004 reported that the presence of glucose help to enchance biofilm formation in *S. aureus.* The presence of glucose induced *rbf* gene in *S. aureus* was responsible for the regulation of multicellular aggregation step of *S. aureus* biofilm formation [18].

The combination of both amylase and protease did not result in 100 % antibiofilm activity (Figs. 5 and 6). The biofilm matrix of *P. aeruginosa* was composed by various components, such as complex polysaccharides, adhesion proteins, rhamnolipid and eDNA [19]. Meanwhile, the biofilm matrix of *S. aureus* consisted of polysaccharide intracellular antigen, eDNA, autolysin and teichoic acid slime layers [20]. The enzyme is substrate-specific, in which case the amylase and protease would only degrade the polysaccharide and protein fraction of the biofilm matrix, respectively. Thus, other components such as lipid and eDNA would still remain intact. Hence, explaining why the activity would not reach 100 % because the enzyme had depleted all of the available substrate [14].

Compilation of antibiofilm destruction and inhibition activity data demonstrated that there was no linear correlation between amylase or protease enzyme activities with the antibiofilm activity (Figs. 5 and 6). This may be due to the fact that antibiofilm used in this study was still in crude form and the usage of BHI medium without any inducer also produced high antibiofilm activity. It is possible that there were other components in the extracts other than both enzymes that also contributed to the destruction of the biofilm. CS and CYS crude extracts which had both enzyme activities had lower amylase enzyme activities than BHIS. There is a possibility that this is due to proteolytic attack of protease on amylase [5].

## Conclusions

*Arthrobacter* sp. CW01 produced two enzymes with antibiofilm activities, which were amylase and protease. However, the antibiofilm activity of *Arthrobacter* sp. CW01 was specific to the type of pathogenic bacteria that were inhibited, production media, inducers and concentration of the crude extracts used to treat the biofilm. It was also revealed that the utilization of different production media and inducers resulted in different metabolism which at the end affected the antibiofilm activity. The amylase and protease production were significantly induced by the addition of starch, casein and yeast extract. Nevertheless, the increase in amylase and protease enzyme activities were not always followed by the increase of biofilm destruction or inhibition activities, indicating that both of the enzymes had reached the maximum concentration to optimally destroy or disrupt the biofilm structure.

### Methods

### Antibiofilm producing bacterial strain

*Arthrobacter* sp. CW01 used in this research was obtained from Cunca Wulang River, West Flores, Indonesia. This bacterium was maintained on Glucose Yeast Malt *Streptomyces* (GYMS) agar and incubated at 28 °C for 7 days.

### Biofilm-forming pathogenic bacteria

Two pathogenic bacteria used for antibiofilm assay, *Pseudomonas aeruginosa* (ATCC 27,853) and *Staphylococcus aureus* (ATCC 25,923) were procured from Microbiologics, Inc. Both of the bacteria were cultured on Brain Heart Infusion Agar (Oxoid) at 37 °C, overnight.

### Screening for antibiofilm agent production media

Amylase as antibiofilm agent from *Arthrobacter* sp. CW01 was screened with five production media (Table 13) and then stained with iodine solution.

*Arthrobacter* sp. CW01 was spotted on all of those agar production media and incubated at 28 °C for 7 days. Each agar plate was stained with iodine solution for 5 min. Amylase activities on various media were compared by the size of the hydrolytic zone and calculated with this formula:

Hydrolytic zone = Diameter of the clearance zone – Diameter of the colony [21].

### Production of antibiofilm agents

*Arthrobacter* sp. CW01 was grown on GYMS agar slant at 28 °C for 5 days. After five days, the inoculum in the agar slant was re-suspended with 5 mL of physiological saline. One mL of inoculum was transferred into 50 mL of GYMS broth in 250 mL Erlenmeyer flask and incubated at 28 °C with 120 rpm agitation for 5 days. This served as the seed culture for antibiofilm agent production. One mL of the seed culture was inoculated into 50 mL of each production broth medium in 250 mL Erlenmeyer flask. Each production medium was carried out in triplicate (with the total of 15 flasks). These flasks were incubated at 28 °C with 120 rpm agitation for one week.

After seven days, the cultures were centrifuged at 4 °C and 7800 x g for 10 min. Supernatants were transferred into new centrifuge tube and centrifuged again at 4 °C and 7800 x g for 10 min. Subsequently, the supernatants were filtered through 0.20 μm ultrafiltration membrane (Corning). The clear filtrates are referred to as crude extracts and can be stored at 4 °C for a week or -20 °C for a month.

### Determination of amylase activity

Amylase activity was measured by the release of reducing sugar from soluble starch using 3,5-dinitrosalicylic acid (DNS). The reaction mixture contained 0.5 mL of crude extracts and 1 % soluble starch dissolved in 1 mL sodium phosphate buffer 0.1 M (pH 7.0). The mixture was incubated at 37 °C for 10 min. Subsequently, the reaction was stopped by the addition of 2 mL DNS and boiled for 10 min to develop colour. The absorbance of the mixture was measured at 540 nm. As control, 0.5 mL of crude extracts was first added with 2 mL DNS to inactivate the enzyme. After that, the mixture was added with 1 mL soluble starch solution and boiled for 10 min to develop colour.

The absorbance of the sample and control were calculated using standard curve equation to determine the glucose concentration. Standard curve was made using 0.5 mL D-glucose (150 µg/mL; 300 µg/mL; 450 µg/mL; 600 µg/mL; 750 µg/mL) mixed with 1 mL soluble starch solution. The glucose content of the sample was substracted by the glucose content of the control and used to determine the enzyme activity. One unit of enzyme activity is defined as the amount of amylase releasing reducing sugar equivalent to 1 µmol glucose per minute at 37 °C [23]. Amylase enzyme activity and specific activity were calculated using this formula:

$$\text{UA }\left(\text{I}\text{U/mL}\right)\text{=}\frac{\left[\text{Glucose sample}\right]\text{- }\left[\text{Glucose control}\right]}{\text{Glucose MW * Incubation time}}\text{* DF}$$

$$\text{SA (}\text{I}\text{U/mg)= }\frac{\text{UA (}\text{I}\text{U/mL}\text{)}}{\text{[Total protein] (mg/mL)}}$$

*UA represented Unit Activity of Enzyme, SA represented Specific Activity of Enzyme, DF represented dilution factor, MW represented molecular weight.

### Determination of protease activity

Based on our preliminary study, there were other antibiofilm compounds from *Arthrobacter* sp. CW01 besides amylase which was identified as protease. In this study, protease activity measurement was done by measuring the release of tyrosine from casein hydrolysis using modified Sigma method. The reaction mixture for sample analysis contained 200 µL of crude extracts and 0.65 % (w/v) casein dissolved in 800 µL of sodium phosphate buffer 0.1 M (pH 7.0). The mixture was incubated at 37 °C for 10 min. Subsequently, the reaction was stopped by the addition of 500 µL of trichloroacetic acid 0.1 M. As control, 800 µL casein was incubated at 37 °C for 10 min and then was added first with 500 µL trichloroacetic acid 0.1 M. Subsequently, the control mixture was added with 200 µL of crude extracts.

Both sample and control had the same treatment from this step onwards. The sample and control were incubated at room temperature for 30 min. After that, each of them was centrifuged at 13,000 rpm for 10 min. 400 µL of the supernatant was transferred into new microtube. One mL of Na_2_CO_3_ 0.4 M and 200 µL of Folin-Ciocalteu reagent were added into the mixture. Each microtube was incubated at 37 °C for 30 min. Thereafter, the microtubes were centrifuged at 13,000 rpm for 10 min. The absorbance of the supernatant was measured at 655 nm and calculated using tyrosine standard curve equation. Tyrosine concentration of the sample was reduced by the tyrosine concentration of the control and used to determine the enzyme activity. One unit of protease activity is defined as the amount of enzyme that is needed to hydrolyze casein to produce 1 µmol of tyrosine product per minute at 37 °C. Protease enzyme activity and specific activity were calculated using this formula:

$$\text{UA}\left(\text{I}\text{U/mL}\right)\text{=}\frac{\left[\text{Tyrosine sample}\right]\text{-}\left[\text{Tyrosine control}\right]}{\text{Tyrosine MW * Incubation time}}\text{* DF}$$

$$\text{SA (}\text{I}\text{U/mg)= }\frac{\text{UA (}\text{I}\text{U/mL}\text{)}}{\text{[Total protein] (mg/mL)}}$$

*UA represented Unit Activity of Enzyme, SA represented Specific Activity of Enzyme, DF represented dilution factor, MW represented molecular weight.

### Determination of protein content

Protein content of the crude enzyme was measured with Bradford method. The reaction was carried out by adding 0.4 mL of crude extracts with 8 mL of Bradford reagent and mixed thoroughly. This mixture was incubated for 5 min in room temperature. Blank was prepared by mixing 0.4 mL of deionized water with 8 mL of Bradford reagent. The absorbance of the crude enzyme was determined by using spectrophotometer (λ = 595 nm). Total concentration of the protein was calculated using standard curve equation of bovine serum albumin.

### Antibacterial activity test

Antibacterial activity test was carried out using agar diffusion test (Kirby-Bauer method). Two pathogenic bacteria, *P. aeruginosa* (ATCC 27,853) and *S. aureus* (ATCC 25,923) were grown on BHI (Oxoid) until a turbidity of 0.5 McFarland was reached. The cultures were uniformly spread over the surface of Mueller Hinton Agar (Oxoid) plate using sterile cotton swab. Excess moisture was allowed to dry for 10 min. The sterile blank antimicrobial susceptibility discs (Oxoid) were placed over the swabbed plates and 10 µL of the crude extracts were loaded on the discs. The plates were incubated at 37 °C for 24 h and the diameter of inhibition zone is measured [24].

### Biofilm eradication activity assay

Biofilm eradication activity assay was performed with static biofilm. Bacterial pathogens were grown in BHI (Oxoid) supplemented with 1 % D-glucose and then incubated at 37 °C until the absorbance value reached 0.132 (λ = 600 nm). Each well in 96-well microplates (Iwaki) was filled with 200 µL of cultures and incubated at 37 °C, overnight. Mature biofilm were then mixed with 5 % (v/v), 10 % (v/v), 15 % (v/v), and 20 % (v/v) of crude extracts from every production treatment and incubated at 37 °C for 30 min. After incubation, planktonic cells and the media were discarded. Adherent cells were rinsed twice using distilled water and air dried. These adherent cells supposed to be the biofilm, were stained with 0.4 % crystal violet and incubated for 30 min. Dyes were discarded and washed with distilled water for 5 times and then air dried. The stained biofilm were solubilized with 200 µL of 96 % ethanol for 5 min and then transferred into new microplate. The optical density of the solution in each well will be determined at 595 nm using microplate reader [25].

### Biofilm inhibition activity assay

Bacterial pathogens were grown in BHI (Oxoid) supplemented with 1 % D-glucose and incubated at 37 °C until the absorbance value reached 0.132 (λ = 600 nm). Each well in 96-well microplates (Iwaki) were filled with 200 µL of pathogenic bacteria cultures along with 5 % (v/v), 10 % (v/v), 15 % (v/v) and 20 % (v/v) crude extracts from every production treatment. Microplates were incubated at 37 °C, overnight and assayed on the next day [25].

### Determination of antibiofilm compound activity

Percentages of antibiofilm eradication and inhibition activity were calculated using this formula [25]:

$$\text{\% }\text{Activit}\text{y= }\text{1-}\frac{\text{OD sample-OD negative control}}{\text{OD positive control-OD negative control}}\text{*}\text{100\%}$$

*Positive control contained pathogenic bacteria without crude extracts, negative control contained broth medium without crude extracts, blank contained broth with crude extracts.

### Statistical analysis

Enzyme and antibiofilm activities were analyzed using one-way analysis of variance (ANOVA). Antibiofilm activities were tested further with Tukey’s Honest Significant Difference (HSD) Test to seek the significant differences between specific groups. Every treatment was repeated 9 times and analyses were performed with 95 % confidence interval using IBM SPSS Statistics 24.

## Limitation

This research did not identify specifically what are the molecules of the antibiofilm agents, only identifying them generally as amylase and protease.

and/or analysed during the current study are available in the [GenBank] repository with the accession number JX434848 for DNA sequencing of *Arthrobacter* CW01.

## Data Availability

The datasets generated.
